# Multilesional pyoderma gangrenosum secondary to Hodgkin lymphoma

**DOI:** 10.1016/j.jdcr.2026.01.013

**Published:** 2026-01-20

**Authors:** Kendall McDaniel, Simrithaa Karunakaran, Laura Upton, Laura Russell, Gillian Heinecke, Nicole Burkemper

**Affiliations:** Department of Dermatology, Saint Louis University School of Medicine, St. Louis, Missouri

**Keywords:** dermatology, hematologic malignancy, Hodgkin lymphoma, pyoderma gangrenosum

## Introduction

Pyoderma gangrenosum (PG) represents a rare cutaneous inflammatory condition characterized by rapidly expanding ulcerations. While it is associated with underlying systemic diseases, primarily autoimmune disorders such as inflammatory bowel disease and rheumatoid arthritis, the relationship between PG and various hematologic malignancies is less defined.^1^ Herein, we present a case of multilesional PG in a patient with newly diagnosed Hodgkin lymphoma.

## Case report

A 67-year-old male presented to an outside hospital with a 3-month history of multiple cutaneous ulcerations associated with weight loss, night sweats, and chronic cough. Skin lesions began as painless nodules that rapidly ulcerated and progressed to involve his face, back, abdomen, and extremities. He was previously evaluated at an outside hospital and underwent extensive infectious workup including blood tests for HIV, syphilis, monkeypox, bartonella, histoplasmosis, blastomycosis, aspergillosis, tuberculosis, and blood and wound cultures, all of which were negative or nonreactive. Computed tomography of the chest revealed mediastinal lymphadenopathy and a right lower lobe pulmonary mass concerning for malignancy. Skin biopsy findings were consistent with PG. The patient was transferred for further inpatient workup.

**Fig 1:**
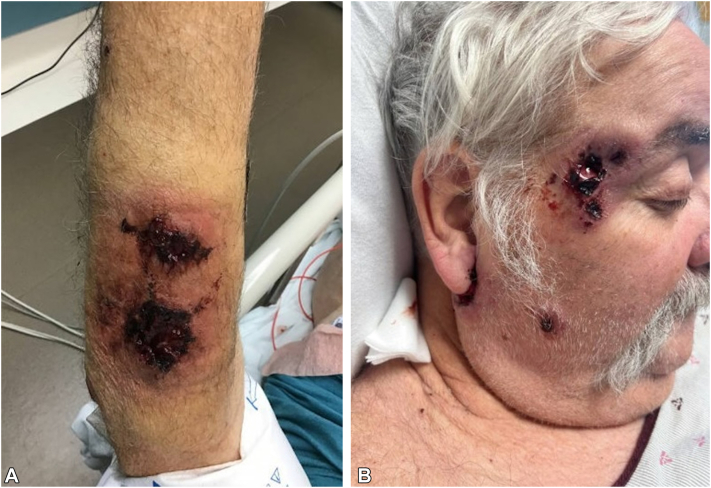
Initial dermatologic evaluation. Hemorrhagic ulcerations with undermined, violaceous borders involving the **(A)** right dorsal forearm and **(B)** right temple.

**Fig 2:**
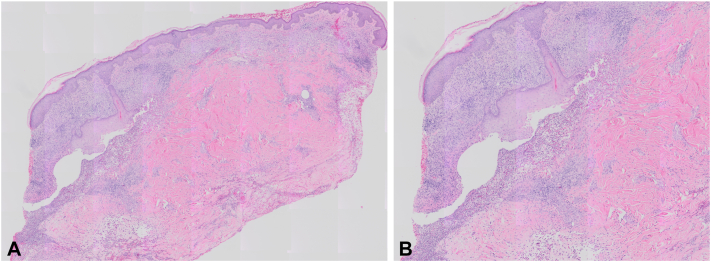
Hematoxylin and eosin (H&E). Intradermal neutrophilic abscess with fragments of necrotic tissue **(A)** low magnification and **(B)** high magnification.

On presentation, physical examination demonstrated well-circumscribed hemorrhagic ulcerations with undermined, violaceous borders involving bilateral temples, right postauricular region, upper abdomen, bilateral forearms, and bilateral distal lower extremities (Fig 1). Punch biopsies were obtained from the right neck and right medial brow, which demonstrated abscess with dermal necrosis and a dense dermal neutrophilic infiltrate (Fig 2). Grocott’s methenamine silver and gram stain were negative for fungal elements and bacteria, respectively. Staining with PAX-5 revealed only few B-cells in the infiltrate. Tissue culture was negative for bacteria, fungi, and acid-fast bacilli. Computed tomography of the chest, abdomen, and pelvis was notable for diffuse lymphadenopathy, a cavitary lesion in the right lower lobe of the lung, and splenomegaly. Subsequent left supraclavicular lymph node biopsy confirmed classic Hodgkin lymphoma, nodular sclerosis type. The hematology-oncology service was consulted for further staging and management.

The patient was treated with intralesional triamcinolone, clobetasol, prednisone 80 mg, and dapsone 25 mg (anemia prevented higher doses of this). He underwent 1 cycle of chemotherapy with doxorubicin, vinblastine, and dacarbazine (AVD) during admission. At a follow-up of 1 week after discharge, the patient was noted to have significant improvement in skin lesions. Steroids were weaned and dapsone dosing was decreased to every other day. The patient was briefly readmitted for sepsis in the setting of cellulitis and Serratia bacteremia. At that point, due to concern for myelosuppression associated with AVD, the patient was switched to single-agent brentuximab vedotin that was subsequently re-escalated to AVD therapy. His ulcers continue to heal and have not recurred.

## Discussion

Although the etiology of PG is not fully understood, it is thought to involve an overexpression of proinflammatory cytokines resulting in auto-inflammation, impaired balance of regulatory T cells and T helper 17 cells, and abnormal neutrophil chemotaxis.^2^ PG may occur independently, as part of an autoinflammatory syndrome or in association with systemic disease.^2^^,^^3^ Studies have shown that more than 50% of PG cases are associated with an underlying comorbidity, particularly inflammatory bowel disease, inflammatory arthritis, and hematologic malignancies.^1^^,^^2^^,^^4^ While the prevalence of hematologic malignancy in PG has been documented in a general range of 3.9% to 45.6%,^1^ a literature review comprised of 823 patients reported that 12.5% of cases were associated with hematologic disorders,^4^ most frequently myelodysplastic syndrome followed by monoclonal gammopathy of undetermined significance and acute myeloid leukemia.^1^^,^^4^ However, an association between PG and Hodgkin lymphoma has not been well established. Due to the rarity of PG, there is a paucity of data stratifying its association with the broad spectrum of hematologic malignancies, particularly lymphomas.

The case presented above illustrates a patient initially presenting with PG and subsequently diagnosed with nodular sclerosis type classical Hodgkin lymphoma. Cutaneous ulcerations were multifocal in nature and preceded by painless nodules. While PG has historically been considered a diagnosis of exclusion, the patient’s clinical and pathologic findings were largely consistent with PG. It is important to note that symptoms rapidly improved following initiation of systemic corticosteroids, dapsone, and chemotherapeutic agents without development of new skin lesions.

One study that analyzed PG characteristics across hematologic malignancies found that ulcerative PG was the most common subtype and that anatomic involvement was most commonly multifocal (34%) or localized to the lower extremity (33%).^1^ Studies have shown that hematologic malignancy confers a 4-fold to 6-fold increased risk of in-hospital mortality and an increased utilization of health care resources.^5^ Nonetheless, data remain limited regarding how clinical presentation and prognosis differ by specific hematologic malignancy type.

## Conflicts of interest

None disclosed.
